# Remarks on Italian Medicine

**Published:** 1844-04

**Authors:** 


*Remarks on Italian Medicine.*—An intelligent French physician, M. Combes, has recently paid a visit to Italy, with the view of examining into the state of medical doctrine and practice in some of the leading schools of that interesting country. We have not yet seen the original work, but, meeting with a copious review of it in one of the Paris Journals, we deemed it worth while to make the following extracts from it.

"Hippocrates has truly observed that 'in general whatever the soil of a country produces is conformable to the country itself.' This remark is quite as applicable to the inhabitants, as it is to the vegetable productions, of a country. The character and temperament of man are in no slight degree influenced by the medium in which he is born and developed; his bodily health, as well as the passions of his soul and the faculties of his mind, are all more or less under the control of the physical circumstances in which he is placed. If he is the king of nature, his sovereignty resembles that, not of an arbitrary or despotic, but of a constitutional throne, hemmed in by charters and by parliaments; and the power that he wields is ever under the weight of legal restrictions. How little suffices to alter the character and the energies of human beings! A few additional degrees of temperature, the exposure or non-exposure to a certain wind, the nature of the soil on which they live—these, not to mention many other circumstances, are found to modify not only the characters and manners of a people, but even the vigor and the power of their intellect. You aspire to the glories of science, and the atmosphere which you breathe condemns you to become an artist; your mind dreams on discoveries of practical philosophy, and pants to tread the path of calm observation, and you find that, in spite of all your wishes, your imagination cannot resist the allurements of nature around you, but plunges into the fairy domains of ideality and romance.

"Under whatever clime human beings come into this world, they all bring with them at birth the same constitution of moral development; the same faculties lie dormant in the souls of all. These, indeed, are unequal from the first in different persons, in consequence of certain hereditary elements which stamp a peculiarity on each nation, each family, and each individual; and this inequality will be more and more strongly marked according to the influences of climate and of early education. This is the reason why nations, although possessing most of the attributes of intellectual greatness, are nevertheless distinguished from each other by the special development of one faculty in a predominant degree. In all ages have the Greeks been pre-eminent for the fervor of their imagination, and the inhabitants of Britain for their cold and prudential reason.

"Nations, like individuals, have therefore their characteristic temperaments or idiosyncrasies of constitution. Now the temperament of Italy is its ideality; its very existence is eminently artistic. Living in a region clothed with light (*vestitos lumine colles*), bathed in a warm and balsamic air, and breathing an atmosphere of romantic associations, little is the wonder that its inhabitants are so distinguished from almost every other people for the richness and grace of their sensual conceptions. Everything in nature around them leads the mind to indulge in images of softness and beauty, and to withdraw it from abstruse and elaborate studies. Now what is the result of this tendency in reference to scientific pursuits? In schools, where pupils and masters alike have all more of the poetic temperament than they are themselves aware of, the character of the instruction will certainly be more or less dogmatic; and why?—because implicit faith is a positive *besoin* of such a mental organization. Such a state of mind naturally leads on to a taste for generalizing on all subjects, and this very tendency is promoted by the copious richness of their sonorous language. Thus it is that the systematic conceptions of the Italians, proceeding from deep conviction, lead to exaggerated applications in theorizing—and often, too, to an inordinate rashness in practice.

"The characteristic features of French medicine in the present day are a sort of disrelish for all systems, and a taste for the elaborate investigation of every problem of inquiry. Hence the practice of rigorous and oft-repeated observation, the numerous experiments, and the constant recourse to the aids of the scalpel, the test-tube, and the microscope. Indicative of the same thing is the subdivision of science into a number of different branches. Each of these is pursued with indefatigable zeal by its different votaries.

"It is scarcely necessary to allude to the characteristics of German medicine: the same laborious curiosity and indefatigable research, the same fondness for mystical speculation, the love of the marvellous and extravagant in physics as well as in metaphysics—these long have been, and still are, the prominent features of the German school."

In Italy at present (according to the observations of M.

Combes) there are very few men who apply themselves with diligence to the patient investigation of details upon any particular subject of inquiry. Most of the physicians have adopted certain general principles of medical doctrine, and on these they base their practice. Hence it is that almost all their treatises assume the synthetic form, and that, even in their works of elementary instruction, the exposition and description of details are too often sacrificed to the abstract generalities of an ingenious dogmatism.

But let it not be forgotten that we are talking of a country which has produced a Spallanzani, a Morgagni, a Scarpa, &c., and can still boast of Bellingeri, not to mention other distingnished names of medical literature. Yet, in spite of these eminent exceptions, it cannot be denied that many of the branches ef professional knowledge have made but little progress, for very many years, on the other side of the Alps.

Physiology has retained pretty generally a purely dynamic character; the classical work of *Medici* does not even mention a vast number of well established facts, and the same remark holds true of Professor Martini at Turin. If such be the state of physiology, or that branch of medical science that treats of the functions of the body in a state of health, we cannot be surprised that those of pathology and of clinical medicine are very faulty and defective in many respects.

"In some districts of Italy, and especially at Naples and Modena, Hippocratism is altogether prevalent. In the latter city, indeed, the Faculty of Medicine may be considered as an association less for instruction in modern medicine than for the perpetuating the doctrines of the Coan sage. The orthodoxy of the school is based upon them; everything is redolent of antiquity; the students are taught to regard the Aphorisms as the oracles of medical truth; and when they aspire to the doctorate, they are called upon to expound and illustrate one or more of these pithy sayings; and it is with their hands laid upon the works of the old Greek that they take the oath upon the day of their inauguration.

"While the doctrines of the Hippocratic medicine, more or less liberally interpreted, are almost universally received through the whole of the south of Italy, the northern half of the Peninsula has been long agitated by the disputed tenets of Contra-stimulism. Turin, Pavia, and especially Milan, are at the head-quarters of this school. Rasori, its founder, started, it is well known, as a disciple of the Brunonian doctrines, and was the translator of the Scotch physician's work. All the phenomena of disease he strove to reduce to one simple morbid action, viz: excessive or deficient stimulation; and like every other advocate of an exclusive system of pathology, he fell into the most egregious blunders, which proved to be injurious alike to sound theory and to safe practice. All diseases he considered to be systematic or general; and local inflammations therefore he looked upon as mere complications or accidental phenomena—the very reverse of the Broussaian creed. Professor Tommasini, of Parma, adopted a sort of intermediate doctrine between these conflicting systems. Assenting to the general principle of his fellow-countryman Rasori, he was too sagacious not to observe that local irritation, when extensive or long continued, is apt to give rise to constitutional diseases. Let us not deprive this distinguished physician of his due; it is right to acknowledge that he clearly enunciated the importance of local affections, as the cause of many diseases, before Broussais had published a line upon the subject.

"One of the most eminent physicians of Italy in the present day is M. Buffalini of Florence. His clinical lectures draw a large concourse of students, and his fame nearly equals that of his distinguished rival of Parma, Tommasini. He is generally regarded as 'un medicin organicien;' and yet his lectures and practice savour strongly of the Essentialist school. In commenting upon any case of fever, he pays minute attention to every appreciable alteration alike of the fluids and of the solids, and he does not hesitate to acknowledge openly that the changes observable, either in one or in the other, are by no means uniformly in accordance with the severity of the constitutional affection, or can be considered as the proper *causes* of it. In many respects he is a decided Humoralist; but while leaning strongly to the opinion that most fevers are attributable to certain changes in the circulating fluid, he on the whole does not trouble himself much with prying into the nature of morbid individualities, but contents himself with scrutinizing their exciting causes, the various symptoms which they exhibit, and with devising the means best fitted for their relief. Every one, who has had an opportunity of witnessing his practice, speaks in high terms of it: avoiding the pernicious errors of Contra-stimulism, (which he has contributed in no small degree to throw into discredit,) he shows great discretion and tact in the use of his remedies. He has published an able work entitled '*Des fondemens de la Pathologie Analytique.*'

"Italy is far behind France in many of the departments of medical science, and certainly in none more so than in chemistry. There are no good laboratories for the preparation of medicines, in almost any part of the country; and hence little or no progress has been made in toxicology, although the writings of M. Orfila have been long highly appreciated. What will probably surprise most travellers, as much as anything, is to find that no correct analysis of the numerous mineral waters of Italy has been made by any native physician.

"Italy possesses more journals of medicine and pharmacy than France does; (we certainly were not prepared for this announcement); but the division of the country into so many separate states places a great bar to their diffusion and general usefulness. The medical press suffers much from the consequences of the general political organism of the country; it has not a few obstacles to overcome, and a good many dangers to avoid. Thus, for example, when Homœopathy first made its appearance at Milan, under the patronage of the Austrian Government, the subject, it was soon found, could not be discussed in all its bearings with perfect freedom. The whole business of journalism is fettered and cramped by restrictions and vexatious annoyances. Hence it is that scarcely any of the periodicals bear that stamp of originality and freedom of opinion, which constitute the main utility of such publications; and that their chief contents are mere compilations from the French, English, and German journals. As long as such a state of things lasts, we cannot look for that energy and devotedness in the cause of professional advancement which we find in the writers of France, Germany, and Great Britain. Surely medical science may fairly be regarded as a free and open field for unlimited boldness of speech and inquiry; but alas! it has always been found that tyranny is never satisfied until it has brought everything under its iron yoke."—*Bulletin of Medcal Science—Gazette Medicale.*

